# Development, implementation, and results of a simulation-based hands-on brachytherapy workshop for medical students

**DOI:** 10.1007/s00066-023-02058-w

**Published:** 2023-03-07

**Authors:** Matthias A. Mäurer, Sonia Drozdz, Juliet Ehrenpfordt, Michael Schwedas, Melissa Friedlein, Nadine Hille, Cora Riede, Steffen Schrott, Maximilian Graf, Georg Wurschi, Marcel A. Kamp, Andrea Wittig, Stefan Knippen

**Affiliations:** 1 Department for Radiotherapy and Radiation Oncology, University Hospital Jena, Friedrich-Schiller-University, Bachstr. 18, 07743 Jena, Germany; 2 Clinician Scientist Program “OrganAge”, Jena University Hospital, 07747 Jena, Germany; 3 Clinician Scientist Program “CSP-11”, Jena University Hospital, 07747 Jena, Germany; 4 Department of Neurosurgery, University Hospital Jena, Friedrich-Schiller-University, Jena, Germany

**Keywords:** Medical education, Simulation-based training, Multicatheter brachytherapy, Accelerated partial breast irradiation, Early-stage breast cancer

## Abstract

**Purpose:**

The new Medical Licensing Regulations 2025 (*Ärztliche Approbationsordnung*, ÄApprO) require the development of competence-oriented teaching formats. In addition, there is a great need for high-quality teaching in the field of radiation oncology, which manifests itself already during medical school. For this reason, we developed a simulation-based, hands-on medical education format to teach competency in performing accelerated partial breast irradiation (APBI) with interstitial multicatheter brachytherapy for early breast cancer. In addition, we designed realistic breast models suitable for teaching both palpation of the female breast and implantation of brachytherapy catheters.

**Methods:**

From June 2021 to July 2022, 70 medical students took part in the hands-on brachytherapy workshop. After a propaedeutic introduction, the participants simulated the implantation of single-lead catheters under supervision using the silicone-based breast models. Correct catheter placement was subsequently assessed by CT scans. Participants rated their skills before and after the workshop on a six-point Likert scale in a standardized questionnaire.

**Results:**

Participants significantly improved their knowledge-based and practical skills on APBI in all items as assessed by a standardized questionnaire (mean sum score 42.4 before and 16.0 after the course, *p* < 0.001). The majority of respondents fully agreed that the workshop increased their interest in brachytherapy (mean 1.15, standard deviation [SD] 0.40 on the six-point Likert scale). The silicone-based breast model was found to be suitable for achieving the previously defined learning objectives (1.19, SD 0.47). The learning atmosphere and didactic quality were rated particularly well (mean 1.07, SD 0.26 and 1.13, SD 0.3 on the six-point Likert scale).

**Conclusion:**

The simulation-based medical education course for multicatheter brachytherapy can improve self-assessed technical competence. Residency programs should provide resources for this essential component of radiation oncology. This course is exemplary for the development of innovative practical and competence-based teaching formats to meet the current reforms in medical education.

## Introduction

The further development of medical studies within the framework of the new Medical Licensing Regulations 2025 includes an orientation from fact-based to competence-based learning and focuses on practical, longitudinal, and interdisciplinary training [1]. The specialty of radiation oncology and radiation therapy, as an integral part of therapeutic oncology, represents an interdisciplinary interface and should be adequately addressed in medical education because of its public health importance [2, 3]. Consequently, there is a great need for high-quality teaching and training in radiation oncology to continue during medical school as well as during residency training [4–8].

In this regard, structured training in brachytherapy techniques is particularly suited to teach practical radiation oncology skills. To date, such courses are rarely offered, although they provide graduates with more confidence and experience in the use of the method [9].

For example, training workshops using a phantom-based simulator have been established for brachytherapy of prostate cancer [10]. In addition to teaching ultrasound-guided treatment planning and implantation techniques, the CT simulation images allow subsequent calculation of the resulting spatial dose distribution, thus providing participants with direct feedback on the quality of catheter positioning [11, 12].

For multicatheter brachytherapy in breast cancer, there are no published results of simulation-based workshops in medical education. Therefore, the aim of the present work was to develop a feasible model for teaching and training practical skills in multicatheter brachytherapy of the breast.

Accelerated partial breast irradiation (APBI) using multicatheter interstitial brachytherapy is a highly effective treatment option for early breast cancer [13–15]. The treatment is characterized by precise delivery of a high dose to the target volume while minimizing the dose to organs at risk [16]. However, despite the advantages of this technique, many authors report a decline in brachytherapy due in part to lack of time and inadequate education and training [17].

## Materials and methods

We have (1) developed a new breast model that enables the acquisition of practical skills in multicatheter brachytherapy of the breast and (2) evaluated its feasibility in an education workshop teaching and training students in brachytherapy techniques.

### Breast model

**Fig. 1:**
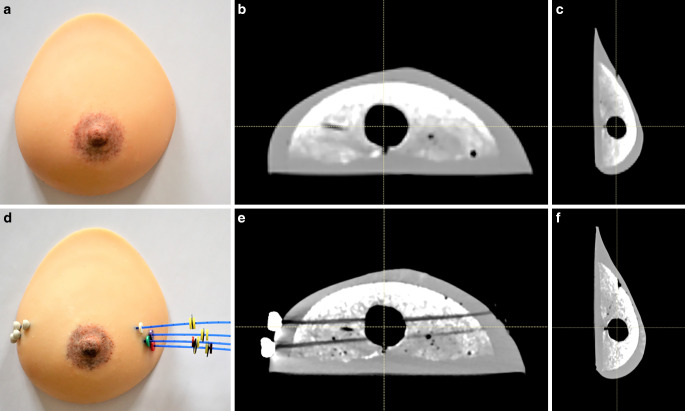
Photographic images (**a**) and CT scans of the breast model in axial (**b**) and sagittal view (**c**). The imitation of the tumor bed appears hypointense on CT. The catheter positions (**d**) within the breast model are visualized by CT scan (**e,** **f**)

For the simulation workshop, a silicone breast model consisting of a soft inner core and an outer skin was used. Therefore, silicone basis component and catalyzation component (SF00-RTV2 Silicone [silicone rubber], SILIKONFABRIK.DE) were mixed in a ratio of 1:1, then silicone oil (SF-V50, SILIKONFABRIK.DE) and Alginat (Protesil ®, ISO 21563) were added. Finally, a color paste (European‑3, FPHE3; Silikonfabrik.de, Ahrensburg, Deutschland) was used to give the model an authentic skin-colored tone. To ensure the soft texture of the inner core, salt and semolina were added to the mixture. The prepared inner core mixture was cast in a ceramic mold with a wire-fixed Styrodur® sphere of approximately 2 cm in diameter, which was supposed to simulate the tumor bed (Fig. 1). After the silicone had cured, the inner core was transferred to a breast-shaped plaster mold treated with silicone removal agent by keeping a gap of 1.5 cm between the inner core and mold, which was filled with the silicone mixture for the outer skin. After a second curing period, the whole breast could be gently removed from the mold.

The breast model was evaluated by experienced radiation oncologists (MM, SD, SK) for its condition, safety, and suitability for training purposes prior to use.

### Applicators

For the teaching course, we used stainless steel needles, 350 mm blind-ended flexible implant tubes, and fixation buttons, which were all sponsored for this workshop by the manufacturer (Varian, Palo Alto, CA, USA).

### CT imaging of the breast cancer model

Imaging of the newly developed breast cancer model using CT scan visualized simulation of the hypointense tumor bed, normal breast tissue, skin, and subcutaneous adipose tissue (Fig. 1).

### Design and learning objectives

The *training course *was aimed at medical students in advanced clinical training (6th–10th semesters) at the beginning of their training. The event was conducted in each case by two radiation oncologists and a student assistant.

We defined the following learning objectives for our hands-on workshop: trainees should be enabled to

- correctly perform the clinical examination of the female breast, localize the tumor bed, and describe clinical findings in technical terms;
- describe the principles of brachytherapy and its areas of application in breast cancer in consideration of alternative radiation techniques;
- perform correctly the implantation of single-leader catheters in predefined positions for breast brachytherapy;
- assess the accuracy of the catheter position a) relative to the tumor bed and b) relative to each other;
- describe the target volume for breast cancer brachytherapy; and
- evaluate the actual implanted catheter position in terms of the resulting dose distribution.

### Workflow of the training course

**Fig. 2:**
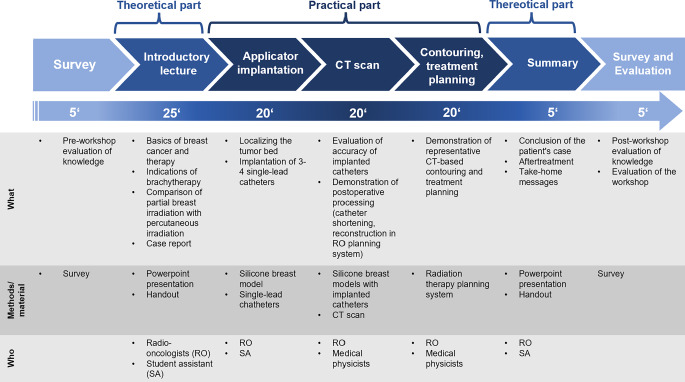
Workflow of the multiprofessional training course teaching practical skills in brachytherapy for early-stage breast cancer

**Fig. 3:**
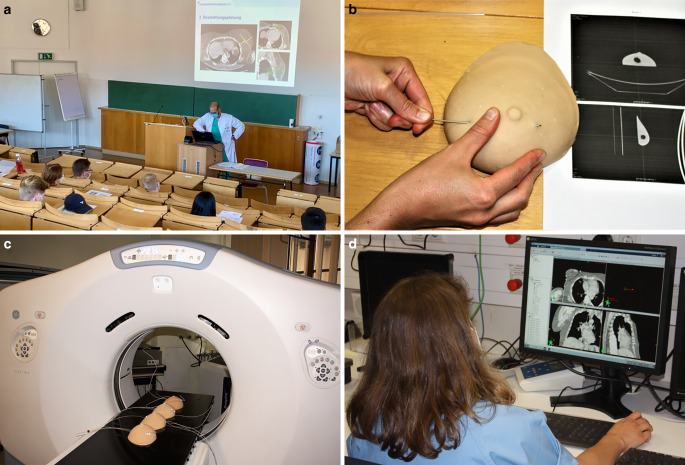
Training course on breast cancer brachytherapy using the newly developed breast cancer model. Following an introductory lecture (**a**), residents and participants trained the implantation of single-lead catheters into the silicone breast model (**b**). CT scan evaluated the accuracy of applicator placement and CT-based contouring and treatment planning was demonstrated and trained (**c**, **d**)

Before the start of the course, participants were asked to fill out a questionnaire intended to assess their knowledge level (Table 1). The course was divided into a 30-minute propaedeutic introduction, including a definition of the learning objectives, and the 60-minute practical part, which consisted of three hands-on stations (Figs. 2 and 3). After the course, the participants were asked again to answer the questionnaire to track their knowledge progress, including a course evaluation.

**Table 1:** Learning progress as illustrated by the mean Likert scale scores and before and after the course

	Before workshop		After workshop	
Survey statement	Mean	SEM	Mean	SEM
I am able to explain basics of etiology and diagnostics of early-stage breast cancer	4.0	0.15	1.8	0.08
I am able to explain the guideline-based therapy of early-stage breast cancer	4.8	0.15	2.0	0.1
I know the different application fields of brachytherapy	4.0	0.18	1.6	0.07
I am able to characterize the patient population suitable for a partial breast irradiation	5.0	0.14	1.8	0.09
I am able to describe the treatment procedure of brachytherapy	4.6	0.16	1.6	0.08
I am able to compare brachytherapy with percutaneous irradiation in terms of advantages and disadvantages	4.5	0.17	1.7	0.09
I am able to implant single-leader catheters into a breast model	5.8	0.09	1.9	0.11
I am able to explain the importance of an accurate catheter implantation for the treatment planning	5.0	0.14	1.5	0.08
I am able to describe the procedure of treatment planning and fractionation	4.9	0.16	2.1	0.09
*Statistical analysis*				
Sum score of all nine statement items	42.4	1.03	16.0	0.48
Corresponding average score per statement	4.7	–	1.8	–

*SEM *standard error of the mean

The propaedeutic part included a lecture on the most important aspects relevant to the curriculum regarding diagnostics and standard guideline-guided therapy of early breast cancer, with a focus on the clinical evidence-based benefit of different radiotherapy techniques after breast-conserving therapy.

The indication for brachytherapy was then presented and the patient population eligible for multicatheter brachytherapy for breast carcinoma was characterized. Partial breast irradiation was then compared with percutaneous irradiation in terms of its advantages and disadvantages. The theoretical part was concluded with a clinical case report.

At the first station of the practical part, a clinical examination of the female breast was first demonstrated to the participants using a silicone breast model, which was then performed by the students under supervision.

Step-by-step catheter implantation was then demonstrated by the instructors. Participants were then requested to work in teams of two to implant three to four introducer catheters homogeneously into the tumor bed of the model simulated by a Styrodur® sphere. The models differed with respect to the sphere, requiring thorough prior palpation for localization. Additionally, CT images of the scanned breast models were presented to each team. After each team had implanted 3–4 applicators under constant supervision, all breast models were CT scanned at the second station of the workshop.

Here, participants were able to assess the accuracy of their applicator placement. In addition, postoperative processing, including catheter shortening and reconstruction in the Radiation Therapy Planning System (Eclipse©, Varian, Palo Alto, CA), of single-lead catheters was demonstrated by a medical physics expert.

At the third station, a representative CT-based contouring and treatment plan was explained and adapted live by a medical physicist. The specifics of 3D radiation planning and treatment flow in clinical practice were discussed. Together with the students, the irradiation plan was optimized. In particular, effects of a suboptimal catheter position on dosimetry and radiation therapy planning (under- and overdosing) were discussed.

At the end of the workshop, the reported clinical patient case was revisited and take-home messages were summarized. After the workshop, participants completed the questionnaire again. Finally, all participants received a handout with the key facts conveyed to consolidate the acquired knowledge at home.

### Evaluation of the training workshop

Before and after the workshop, participants were asked to rate their skills and knowledge on APBI using a questionnaire on a six-point Likert scale (1—completely agree, 2—mostly agree, 3—tend to agree, 4—tend to disagree, 5—mostly disagree, 6—disagree completely). In addition, participants were required to complete a questionnaire after the course to evaluate the entire workshop. Optionally, there were two free questions, “What did you like best?” and “What could be improved?” Participation was anonymous.

### Statistics

Statistics were performed by using IBM SPSS Statistics version 26 (IBM Deutschland GmbH, Ehningen, Germany). Only completely fulfilled surveys were included in the statistical analysis. We hypothesized that the workshop enables participants to enhance their knowledge-based competencies on brachytherapy. The participants assessed their skills on a six-point-Likert scale in a questionnaire consisting of nine items. In a descriptive analysis, mean values and standard error (SEM) for each item were compared before and after the workshop (Table 1). To test our hypothesis, sum scores of all items were calculated. A non-parametric statistical hypothesis test was performed by a paired *t*-test. A significance level below *p* = 0.05 was considered as significant.

## Results

### The brachytherapy breast cancer model

The newly developed breast cancer model was rated as realistic by the seniors involved in the publication with many years of experience in brachytherapy of breast cancer.

Trainees described the brachytherapy breast cancer model as suitable for achieving the previously defined learning objectives (1.16, standard deviation [SD] 0.41 on the six-point Likert scale) and successfully learned practical skills of implanting single-leader catheters into the breast model (5.8 to 1.9). The majority of respondents fully agreed that the workshop increased their interest in brachytherapy (1.15, SD 0.40). Moreover, they were able to describe the contouring principles of the target volume, the treatment planning process, and radiation fractionation schemes (4.9 to 2.1).

### Workshop

Between June 2021 and July 2022, a total of 70 trainees participated in 11 sessions of 90 min each. 62 questionnaires were completed in full.

For all learning objectives, participants indicated that their theoretical and practical skills improved significantly as a result of the course (see Likert confidence scores, Table 1). Participants were able to improve their knowledge-based skills about aspects essential for radiation planning, the pre-radiotherapy process (4.0 at 1.8), and treatment (4.8 at 2.0) of early-stage breast cancer. They were able to expand their knowledge about different application fields of brachytherapy in general (4.0 to 1.6). The workshop raised the awareness of which patients could benefit from partial breast irradiation (5.0 to 1.8). Furthermore, the workshop enabled participants to provide patients with information about the treatment procedure (4.6 to 1.6) as well as the advantages and disadvantages in comparison to external beam irradiation techniques (4.5 to 1.7).

For statistical analysis of the described learning progress, we calculated sum scores based on the Likert confidence scores for all nine survey statements. Before the workshop, the mean sum score was 42.4, which corresponds to an average value of 4.7 on the Likert confidence score. After the workshop, the mean sum score was enhanced to 16.0, corresponding to a value of 1.8 on average. The participants underwent a striking learning process by attending this workshop and were able to improve their knowledge-based competencies on brachytherapy for breast cancer significantly (*p* < 0.001, tested by paired *t*-test).

**Fig. 4:**
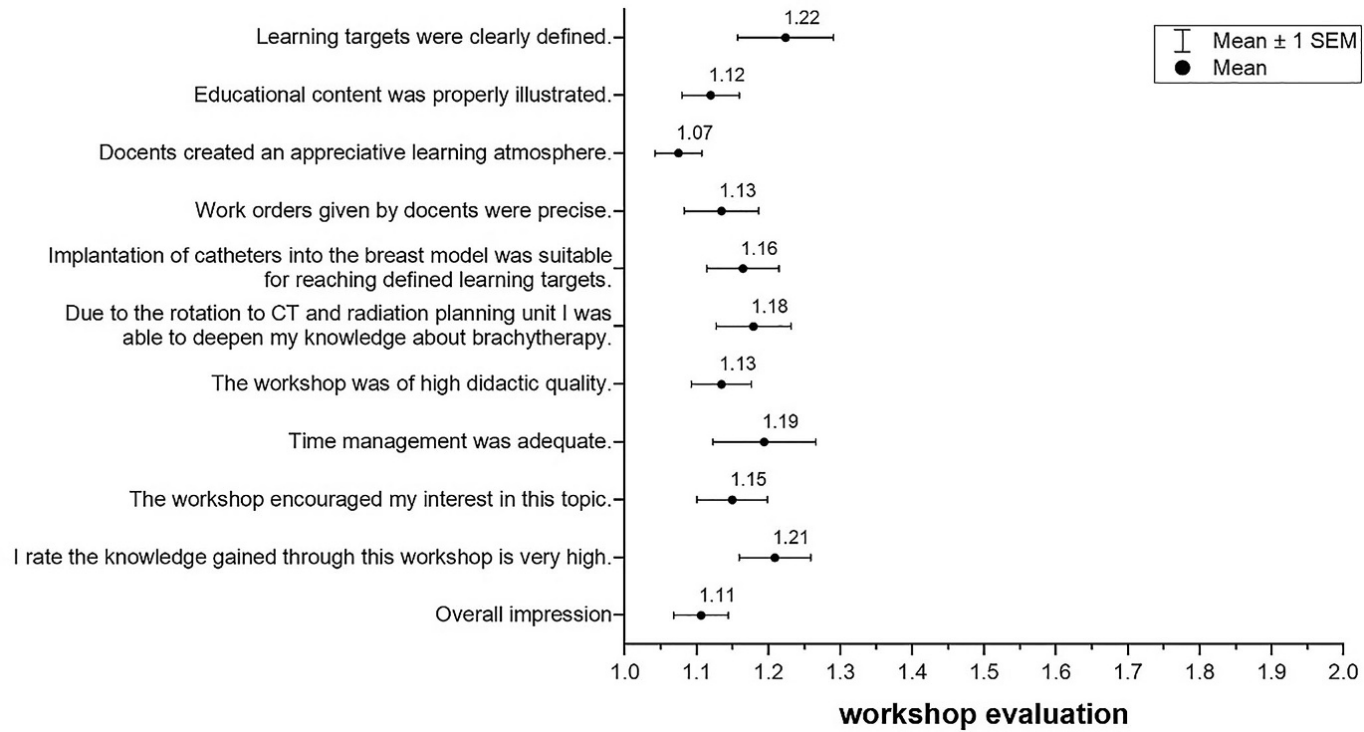
Evaluation of the model and workshop. Items on a six-point-Likert scale (totally agreed–disagreed, and completely/very good–poor for overall impression)

The training workshops were predominantly positive (Fig. 4). The breast model was described as suitable to achieve the previously defined learning objectives (1.16, SD 0.41).

According to the free-text answers, the participants appreciated the practical hands-on session, the well-structured workflow within the real clinical facilities, the appreciative supervision by the lecturer, the color-printed handout, the link between the propaedeutic and practical parts, and the learning and working atmosphere in general.

In the first seminars, the sluggishness of the catheter application due to the high resistance of the model was criticized several times. After optimizing the composition of the breast model, this point of criticism did not reoccur.

## Discussion

This workshop is the first simulation-based hands-on workshop on interstitial multicatheter brachytherapy for breast cancer described in the literature. The simulation of catheter placement on the breast model enabled participants to achieve the predefined learning objectives. According to the self-assessment, participants benefited significantly from an increase in their competency-based knowledge measurable using the six-point Likert scale. Furthermore, the students appreciated the practical hands-on elements, their close integration with the theoretical content, and the insight into real clinical facilities.

The workshop follows a competency-based and interdisciplinary teaching approach. Thus, it is significantly oriented towards the current reform efforts of medical teaching in Germany, which are expressed in the Masterplan Medizinstudium 2020 and in the revision of the National Competence-Based Catalogue of Learning Objectives in Medicine (NKLM). Here, the demand for a consistent practice and competence orientation of teaching with a special focus on an understanding of effective interdisciplinary collaboration between different professions in health care is found. By synthesizing individual learning objectives, more practice-related competencies are to be taught. The acquisition of competencies not only includes pure knowledge aspects, but also requires additional manual and communicative skills in the application of the acquired knowledge. Lectures such as the brachytherapy workshop are particularly suitable for linking cognitive, psychomotor, and affective learning objectives [18–20]. These proclaimed educational principles afford a high potential for developing innovative teaching formats especially in radiation oncology, due to its multiprofessional and interdisciplinary nature [1, 7, 21].

The multiprofessional design of the workshop will provide participants with a profound understanding about brachytherapeutic work and treatment processes as well as interdisciplinary collaboration in radiation oncology. A profound understanding of multiprofessional cooperation in radiation oncology care is instrumental for improved patient outcome and should therefore be further promoted through multiprofessionally designed teaching opportunities [22].

Simulation-based training, based on active learning theories in cognitive and learning sciences, is increasingly demanded for modern medical teaching across disciplines [23]. According to Dale’s Cone of Experiences, active learning processes are significantly more effective than passive ones and allow learners to remember up to 90% of what they learn [24]. Complex cognitive processes result from the interaction between declarative and procedural knowledge. The acquisition of high-quality professional competencies therefore requires the essential integration of practical components into teaching. Especially for medical education, it has been shown that simulation-based teaching concepts are superior to purely theoretical lessons [25, 26]. The positive effects were not only shown with regard to the acquisition of technical knowledge and practical skills, but were even associated with an improvement in patient care, which should be seen as a groundbreaking advantage in view of the intention of medical teaching [27–31]. Furthermore, it has already been demonstrated that simulation-based training achieves particularly sustainable and long-term learning effects [23, 32].

Our results are congruent with observations from other simulation-based workshops on brachytherapy for prostate and gynecologic cancer. Here, it was consistently shown that simulation-based brachytherapy training led to increased confidence in using the method [9, 11, 12]. Despite its advantages, the clinical use of brachytherapy has been declining for years [17]. Conducting such hands-on teaching sessions for residents could be an approach to strengthen the awareness and entrenchment of brachytherapy in radiation oncology.

Recently, it has been reported that there is a great demand for more practical lessons in the training of radiation oncology professionals [33]. While it has been demonstrated that simulation-based education has a high benefit in radiation oncology, it mainly addresses the teaching of contouring. Hence, there is a big gap to fill regarding other specific competencies such like communication or technical skills [34]. Educational training methods like the workshop are a promising approach to meet these needs.

Despite the didactic advantages, only few simulation-based teaching concepts have been established in radiation oncology so far, which is possibly also related to the required personnel, time, and financial burdens. In addition, techniques such as multicatheter brachytherapy of breast carcinoma are not even available at all university centers.

The participants of our workshop particularly appreciated the integration of our workshop into the real clinical setting with participation of the professional groups involved in everyday treatment. They reported being able to better estimate the complexity of the radiotherapeutic treatment chain, with the consequences to be derived from the various treatment steps. From the perspective of cognitive science, the situational learning theory is applied here, according to which learning is understood as a context-dependent process. The content and meaning of what is learned is largely dependent on the environment in which it is taught. Therefore, it is advisable to integrate technical courses into the real-life professional environment [23, 35, 36].

Learning about the various radiotherapeutic procedures, especially interventional techniques such as brachytherapy, could stimulate interest in the entire field of radiation oncology [6, 8]. Although not explicitly recorded in the evaluation form, the majority of students indicated that the workshop increased their interest in the topic.

In addition to the positive results of the workshop described here, some limitations also become apparent. The monocentric nature of this study limits its validity. This is especially true for the questionnaire, which was developed specifically for the study and thus does not represent an established, standardized measurement instrument. Similarly, the number of study participants forms only a small sample size. Furthermore, not all participants completed the questionnaire in full. The voluntary workshop participation of the medical students results in a selection bias, as it is possible that primarily students interested in radiation oncology attended the workshop, which limits a transfer of the results to the general public of medical students. To assess the achievement of the predefined learning objectives, an exam (e.g., as a lightning round or elevator pitch) could be conducted at the end of the event. Likewise, no statement can be made about the long-term learning effect achieved. Further follow-up studies would be necessary for this.

The comparability of the breast model, including the Styrofoam ball as a tumor bed, with a real breast is limited. The consistency of the material was found to be stiffer compared to real breast tissue. A clinical examination of the breast can only be reproduced to a limited extent with the aid of the model, but this was also not the leading intention of this course.

The demand for practice-oriented teaching formats to impart manual skills is countered by the development of an increasing number of digital teaching formats, partly driven by the coronavirus pandemic [37]. While certain elements of the workshop can only be conducted in classroom teaching under supervision (e.g., catheter implantation), some parts are also feasible in digital format. A further development of the course could be the integration of digital technologies as a hybrid format [2]. It would be conceivable to establish a flipped classroom format as preparation for the workshop with the aid of digital teaching materials [38].

The multiprofessional, competence- and practice-based approach of the hands-on workshop is significantly oriented towards the reform movements in the German medical curriculum and could be exemplary for future teaching concepts in radiation oncology.

## Conclusion

The simulation-based medical education course for multicatheter brachytherapy can improve self-assessed technical competence and increase enthusiasm for brachytherapy. Residency programs should provide resources for this essential component of radiation oncology. This course is exemplary for the development of innovative practical and competence-based teaching formats to meet the current reforms of medical education in Germany.
